# Partial Immunoblotting of 2D-Gels: A Novel Method to Identify Post-Translationally Modified Proteins Exemplified for the Myelin Acetylome

**DOI:** 10.3390/proteomes5010003

**Published:** 2017-01-12

**Authors:** Kathrin Kusch, Marina Uecker, Thomas Liepold, Wiebke Möbius, Christian Hoffmann, Heinz Neumann, Hauke B. Werner, Olaf Jahn

**Affiliations:** 1 Department of Neurogenetics, Max-Planck-Institute of Experimental Medicine, 37075 Göttingen, Germany; kusch@em.mpg.de (K.K.); moebius@em.mpg.de (W.M.); hauke@em.mpg.de (H.B.W.); 2 Proteomics Group, Max-Planck-Institute of Experimental Medicine, 37075 Göttingen, Germany; uecker@em.mpg.de (M.U.); liepold@em.mpg.de (T.L.); 3 Center for Nanoscale Microscopy and Molecular Physiology of the Brain, 37075 Göttingen, Germany; 4 Free Floater (Junior) Research Group Applied Synthetic Biology, Georg-August University Göttingen, Institute for Microbiology and Genetics, 37077 Göttingen, Germany; cho@accurion.com (C.H.); heinz.neumann@mpi-dortmund.mpg.de (H.N.)

**Keywords:** post-translational modification (PTM), 2D gel electrophoresis (2DE) blot, myelin, acetylome, SERPA, immunoproteomics, tubulin acetylation, cyclic nucleotide phosphodiesterase (CNP), septin 8 (SEPT8), isoelectric focusing (IEF), immunoblot, cryo immuno-electron microscopy (IEM)

## Abstract

Post-translational modifications (PTMs) play a key role in regulating protein function, yet their identification is technically demanding. Here, we present a straightforward workflow to systematically identify post-translationally modified proteins based on two-dimensional gel electrophoresis. Upon colloidal Coomassie staining the proteins are partially transferred, and the investigated PTMs are immunodetected. This strategy allows tracking back the immunopositive antigens to the corresponding spots on the original gel, from which they are excised and mass spectrometrically identified. Candidate proteins are validated on the same membrane by immunodetection using a second fluorescence channel. We exemplify the power of partial immunoblotting with the identification of lysine-acetylated proteins in myelin, the oligodendroglial membrane that insulates neuronal axons. The excellent consistency of the detected fluorescence signals at all levels allows the differential comparison of PTMs across multiple conditions. Beyond PTM screening, our multi-level workflow can be readily adapted to clinical applications such as identifying auto-immune antigens or host-pathogen interactions.

## 1. Introduction

The regulation of protein abundance and function are key determinants of all vital processes. While modern gel-based and gel-free proteomics provides powerful and easily adaptable methods for comparison of protein abundance, investigation of post-translational protein modifications (PTMs) is still challenging. However, PTMs crucially affect protein activity, interaction, stability and localization, indicating the imperative of understanding PTMs for an understanding of biological function [1].

More than 400 different PTMs are known [1]. In the brain, PTMs are intensely investigated and >5300 PTM sites have been annotated in neuronal proteins from different species (PhosphoSitePlus Protein Modification Resource as of August 2016, http://www.phosphosite.org, [2]). A considerable proportion may be constituted by the brain-specific phosphoproteome [3]. In contrast, for oligodendrocytes, a glial cell type in the central nervous system (CNS), information on PTMs is limited. Oligodendrocytes form myelin to electrically insulate neuronal axons, enabling fast axonal signal propagation. However, the myelin sheath is not only an electrical insulator but may facilitate macromolecule supply for axonal function through cytoplasmic channels [4].

Myelin membranes can be separated biochemically from other cell membranes by discontinuous density gradient centrifugation [5,6]. In the early years of myelin protein investigation on isolated myelin, the protein composition was believed to be of very low complexity [7]. Application of recent gel-based and gel-free proteomic techniques extended the list of proteins identified in CNS myelin to >1000 [8]. Several myelin proteome datasets for CNS myelin are available [5,8,9,10,11,12,13,14,15,16]. However, information on PTMs of myelin proteins and their function remained very limited. Among the classical myelin proteins, functional characterization of PTMs is only available for myelin basic protein (MBP) and myelin proteolipid protein (PLP) [17,18,19,20,21,22,23,24]. Several proteins located in non-compacted compartments of CNS myelin are known to be glycosylated, including myelin oligodendrocyte glycoprotein (MOG), myelin-associated glycoprotein (MAG), oligodendrocyte-myelin glycoprotein (OMgp), and Opalin [25,26].

Screening the myelin proteome datasets revealed a number of enzymes involved in the modification of proteins. Several protein kinases and phosphatases were identified in myelin fractions [16,27]. Other enzymes involved in protein deimination (also referred to as citrullination), glycosylation, methylation, ubiquitinylation, sumoylation and farnesylation were detected in myelin [8,16,27]. At least two lysine deacetylases are abundant, the cytosolic enzymes sirtuin 2 (SIRT2) [15,28,29] and histone deacetylase 11 (HDAC11) [16,27]. However, the target proteins of these deacetylases in myelin remain unknown. For SIRT2, α-tubulin deacetylating activity was described in vitro [30]; yet in *Plp*-deficient mice that lack SIRT2 from myelin, tubulin hyperacetylation in the myelin fraction was not evident [15]. It is likely that these enzymes are relevant for the structure and functions of myelin, but experimental evidence is still pending.

In recent years, remarkable progress in knowledge of PTMs was achieved by mass spectrometry (MS)-based PTM analysis. For phosphoproteomics, this was driven by the development of enrichment strategies, mainly based on immobilized metal ion and metal oxide affinity chromatography [31]. Conversely, such robust chemical enrichment methods are not available for lysine modifications such as acetylation or malonylation, and their analysis usually requires antibody-based enrichment of the modified peptides after enzymatic digestion [32,33,34,35,36]. Alternatively, immunoprecipitation of intact proteins has been applied [37]. Taken together, these techniques use powerful, advanced sample preparation and MS approaches, which are available only in specialized laboratories. Still, for analysis of any new PTMs of interest, time consuming protocol adaptation is required.

For the identification of particular PTMs in gel-based approaches, dedicated staining or labeling methods have been developed including phospho- and glycoprotein-specific stains or click chemistry-based glycoprotein detection [38,39,40,41,42]. Combinations of these methods for concurrent analysis of glycosylated, phosphorylated, and total proteins are possible [39]. 

2DE is a powerful top-down approach to resolve proteoforms including post-translationally modified variants. 2D immunoblotting is thus predestined to be used for targeted PTM screening when specific antibodies are available, as is the case for several PTMs, such as nitrosylation or acetylation [43,44,45]. The major challenge of 2D immunoblot-based screening approaches is the correct matching of the two 2D-gels that are typically run in parallel, one for blotting and immunodetection, and the other for protein staining, excision of spots of interest, and mass spectrometric protein identification. Migration variability between the gels and gel shrinkage during blotting often lead to distorted spot pattern, and may prevent the correct assignment of the protein identity to an immunopositive signal. So far, reliable matching of spot pattern was only possible when a large fraction of proteins was post-translationally modified [43,44], resulting in a high number of “anchor points” on the blotting membrane and thus in the facilitation of the overlay procedure. However, for less abundant PTMs, including acetylated lysine residues in the CNS myelin fraction, reliable overlay was challenging if not impossible in our hands. This motivated us to develop an inexpensive, fast, and comparative 2DE-based method for reliable multiplexing of PTM screening and protein identification, which involves only standard equipment of a biochemical laboratory. The key step of our multi-level workflow are the partial transfer of colloidal Coomassie-stained proteins from 2D-gels to polyvinylidene difluoride (PVDF) membranes, preserving the information on exact spot positions, and the consistent signal detection by near-infrared fluorescence imaging at all levels. Our approach is not limited to a particular type of PTM, but can be easily adapted to any antibody-based PTM screening and extended to other applications such as serological proteome analysis (SERPA) and immunoproteomics [46].

## 2. Materials and Methods 

### 2.1. Animals 

C57BL/6 wildtype mice were bred in the animal facility of the Max-Planck-Institute of Experimental Medicine. For obtaining brain samples, mice were sacrificed by cervical dislocation, brains were dissected, frozen quickly on dry ice and stored at −80 °C until usage. For obtaining optic nerve samples, mice were sacrificed by cervical dislocation, optic nerves were dissected and immersion fixed in 2% (*v*/*v*) glutaraldehyde, 4% (*w*/*v*) formaldehyde in phosphate buffer containing 0.5% (*w*/*v*) NaCl. All experiments were in compliance with the animal policies of the Max-Planck-Institute of Experimental Medicine, approved by the Landesamt für Verbraucherschutz und Lebensmittelsicherheit, the relevant authority for the German Federal State of Lower Saxony. Male mice at an age of 75 days were analyzed.

### 2.2. Myelin Sample Preparation

A light-weight membrane fraction enriched in myelin was purified as described before [47] from mouse brain homogenate in 0.32 M sucrose supplemented with 10 mM nicotinamide, 500 nM TrichostatinA (TSA) for inhibition of deacetylases and with protease inhibitor cocktail (cOmplete, Roche, Mannheim, Germany). The myelin-enriched fraction was resuspended in Tris buffered saline (TBS) (50 mM Tris-HCl, pH 7.4, 150 mM NaCl) supplemented with 10 mM nicotinamide, 500 nM TSA and protease inhibitor cocktail (cOmplete Mini, Roche) and stored at −80 °C. Proteins were delipidated and precipitated by methanol/chloroform according to Wessel and Flügge [48]. The protein pellet was resuspended in 7 M urea, 2 M thiourea, 2% (*w*/*v*) amidosulfobetaine 14 (ASB-14, Serva, Heidelberg, Germany), 30 mM Tris/HCl pH 9 and sonicated three times for 2 min in an ultrasonic bath (Branson 2200, Dietzenbach, Germany) with intermittent incubation at room temperature for 20 min. Protein concentration was determined using the 2D Quant Kit (GE Healthcare, Munich, Germany) according to the manufacturer’s instructions.

### 2.3. Expression and Purification of Lysine-Acetylated Proteins 

By using the genetic code expansion concept [49], *N*-(ε)-acetyl-lysine was site-specifically incorporated into a recombinant protein to be used as process control. For expression of recombinant RAN (rRAN) with N-terminal HisTag, *E. coli* BL21 transformed with pCDF-DUET-PylT-Ran (non-acetylated rRAN) or pBK-AcKRS3 and pCDF-DUET-PylT-RanK71TAG (AcK^90^-rRAN, amino acid numbering according to the sequence of the recombinant protein) were grown, induced and collected essentially as described [49]. For purification of rRAN or AcK^90^-rRAN, cells were incubated in 15 mL phosphate buffered saline (PBS) or PBS with 20 mM nicotinamide, respectively, containing protease inhibitor cocktail (18 µg/mL Pefablock, 0.07 µg/mL Leupeptin, 8.8 µg/mL o-Phenanthrolin, 0.34 µg/mL Pepstatin A), 1 mM dithiothreitol (DTT) and 0.2 mg/mL lysozyme, and lysed by sonication. Extracts were cleared by centrifugation (15 min, 18,000 rpm, 4 °C, JA-30.50Ti). Supernatants were applied to a HisTrap 1 mL FF column using the ÄKTA purifier system with a flow rate of 0.5 mL/min equilibrated with 10 mM Tris/HCl pH 8.0, 200 mM NaCl, 20 mM imidazole. The column was washed with 10 mM Tris/HCl pH 8.0, 200 mM NaCl, 34.4 mM imidazole and bound protein eluted with 10 mM Tris/HCl pH 8.0, 200 mM NaCl, 200 mM imidazole. For the Ni-NTA purification of AcK^90^-rRAN, 5 mM DTT was added to the buffers. rRAN-containing fractions were applied to a HiLoad 26/60 Superdex 75 column equilibrated with gel filtration buffer (50 mM Tris/HCl pH 7.5, 50 mM NaCl, 2 mM Mg(OAc)_2_, 5 mM DTT). Fractions containing rRAN (as analyzed by SDS-PAGE) were pooled, concentrated and stored at −80 °C. Incorporation of AcK was confirmed to be complete by mass spectrometric peptide mapping. Where indicated, 0.5 µg of rRAN and 0.5 µg AcK^90^-rRAN were spiked into myelin samples directly prior to isoelectric focusing.

### 2.4. Isoelectric Focusing

A volume equivalent corresponding to 100 µg myelin protein was mixed with the same volume of rehydration buffer I (7 M urea, 2 M thiourea, 2% (*w*/*v*) ASB-14, 40 mM DTT, 1% (*v*/*v*) Servalyte ampholytes pH 3–10 (Serva)) and solubilized by short sonication and gentle shaking. The sample was filled up to 130 µL with rehydration buffer II (7 M urea, 2 M thiourea, 2% (*w*/*v*) ASB-14, 20 mM DTT, 0.5% (*v*/*v*) Servalyte ampholytes pH 3–10 (Serva)), solubilized as above, centrifuged (2 min 16,000× *g*), and the supernatant subjected to IEF in immobilized pH gradients (IPG BlueStrips, 7 cm, pH 3–10 or 3–12, Serva) in a Protean i12 IEF System (BioRad, Munich, Germany). During active rehydration at 50 V, 20 °C for ~16 h, current was limited to 70 µA. After 6 h active rehydration, paper wigs moisturized with distilled water were placed at both electrode ends underneath the IPG strips. For isoelectric focusing a step-gradient protocol was set according to the manufacturer’s instructions (1: hold at 150 V for 1 h; 2: hold at 300 V for 1 h; 3: ramp to 1000 V in 1 h; 4: ramp to 3000 V in 2 h; 5: hold at 3000 V for 2 h) at 20 °C.

### 2.5. Gel Electrophoresis

Directly after IEF, proteins were reduced in sample buffer (150 mM Tris-HCl, pH 8.5, 2% (*w*/*v*) lithium dodecyl sulfate (LDS), 10% (*v*/*v*) glycerol, 0.51 mM EDTA, 0.22 mM Serva Blue G250, 0.175 mM Phenol Red) containing 50 mM DTT and subsequently alkylated in sample buffer containing 125 mM iodoacetamide (IAA) for 15 min each. For protein separation in the second dimension, IPG strips were placed on precast NuPAGE Novex 4%–12% Bis-Tris ZOOM Protein Gels, 1.0 mm, IPG-well (Thermo Fisher Scientific, Waltham, MA USA), 4 µL Dual Color Protein Standard III (Serva) was used as molecular weight marker and electrophoresis was performed in MES buffer at 180 V. Gels were fixed in 40% (*v*/*v*) ethanol, 10% (*v*/*v*) acetic acid for 60 min, rinsed 3× for 10 min in water and stained with colloidal Coomassie staining solution (0.08% (*w*/*v*) Coomassie Brilliant Blue G-250, 1.6% (*w*/*v*) ortho-phosphoric acid, 8% (*w*/*v*) ammonium sulfate and 10% (*v*/*v*) methanol) over night. Excess dye was removed by washing in water. Gels were imaged using an Odyssey near-infrared scanner (LI-COR, Lincoln, NE, USA) at 700 nm wave length, intensity set to 5.0, quality set to “high” and resolution to 84 µm.

### 2.6. Partial Transfer

Gels stained with colloidal Coomassie were equilibrated twice in MES buffer for 10 min. Proteins were transferred to PVDF membrane (Millipore, Darmstadt, Germany) in transfer buffer (25 mM Bicine, 25 mM Bis-Tris, 1.025 mM EDTA, 10% (*v*/*v*) methanol) in a XCell II Blot Module (Thermo Fisher Scientific) at 30 V for 13 min. Transfer time was empirically optimized for myelin proteins and may need adaptation for other samples and other blotting equipment. After transfer, gels were re-stained with colloidal Coomassie and imaged as described above. All edges of the PVDF membranes were labeled with cross-shaped orientation marks visible in both near-infrared channels to facilitate later overlay of membrane images obtained after colloidal Coomassie staining (700 nm channel) and immunodetection (800 nm channel, see below). A conventional blue laboratory pen (e.g., Lumocolor Permanent, Staedtler, Nürnberg, Germany) served this purpose. Membranes were imaged using an Odyssey near-infrared scanner (LI-COR) at 700 nm wave length, intensity set to 2.0, quality set to “high” and resolution to 84 µm. 

### 2.7. Immunodetection

Coomassie stain was removed by washing the PVDF membranes twice for 5 min in PBS containing 50% (*v*/*v*) ethanol and 0.1% (*v*/*v*) Tween-20. After rinsing briefly in PBS, PVDF membranes were blocked for 30 min in 50% (*v*/*v*) Odyssey Buffer (LI-COR) diluted in PBS. Primary antibodies were applied in 50% (*v*/*v*) Odyssey Buffer (LI-COR), 0.1% (*v*/*v*) Tween-20 in PBS overnight or for two days at 4 °C. Primary antibodies were used as follows: Acetyl Lysine Antibody (1:500, polyclonal rabbit, Immunechem, Burnaby, BC, Canada, IPC0380), Acetyl Lysine Antibody (1:500, polyclonal rabbit, PTM Biolabs, Chicago, IL USA, PTM-105), anti-α-tubulin (1:1000, monoclonal mouse, Sigma, Darmstadt, Germany, T 5168) and anti-HisTag (1:1000, monoclonal mouse, Acris, Herford, Germany, CPA-9028). For Acetyl Lysine Antibodies, incubation for two days improved signal to noise ratio, while overnight incubation was sufficient for all other primary antibodies. PVDF membranes were washed five times for 5 min in 0.1% (*v*/*v*) Tween-20 in PBS. Secondary antibodies diluted in 50% (*v*/*v*) Odyssey Buffer (LI-COR), 0.1% (*v*/*v*) Tween-20 in PBS were applied for 60 min. Secondary antibodies were used as follows: anti-rabbit IRDye 800 (1:5000, polyclonal goat, Biomol, Hamburg, Germany, 611-132-122), anti-mouse IRDye 680 (1:5000, polyclonal goat, Thermo Fisher Scientific, 982289). PVDF membranes were washed four times for 5 min in 0.1% (*v*/*v*) Tween-20 in PBS and once in PBS. Membranes were imaged using an Odyssey near-infrared scanner (LI-COR) at 800 nm (intensity set to 4.5) or 700 nm (intensity set to 5) wave length, quality set to “high” and resolution to 84 µm.

### 2.8. Data Analysis

Original 16 bit scan files were imported into the Delta2D software version 4.3 (Decodon, Greifswald, Germany). Images of Coomassie-stained gels directly after transfer and of the PVDF membrane were warped to the gel image before transfer applying the “exact” mode to the gel image before transfer. All scan files obtained from antibody incubation were warped to the PVDF Coomassie image by overlaying the marks at the edges of the membrane using the “exact” mode. All other composite images were generated using the “implicit” mode. Spots were detected on a fusion gel generated from both Coomassie-stained gel images before transfer and edited manually. Spots were transferred automatically to all original images. Spots with detectable signal in AcK channel and visible Coomassie stain were labeled and excised from the respective Coomassie-stained gel using a punching tool with 1.5 mm diameter.

### 2.9. Protein Identification

Manually excised gel plugs were subjected to an automated platform for the identification of gel-separated proteins [50] as described earlier [15]. Per gel plug, 50 ng of modified sequencing grade trypsin (Serva) stabilized against autolysis were applied. An Ultraflex MALDI-TOF-TOF mass spectrometer (Bruker Daltonik, Bremen, Germany) was used to acquire both peptide mass fingerprint (PMF) and fragment ion spectra, resulting in confident protein identifications based on peptide mass and sequence information. Database searches in the Swiss-Prot primary sequence database (UniProt release 2016_08) restricted to the taxonomy *mus musculus* (16,812 entries including the manually added sequence of rRAN) were performed using the MASCOT Software version 2.3.02 (Matrix Science, London, UK). Carboxamidomethylation of Cys residues was specified as fixed and oxidation of Met residues as variable modifications. Trypsin was specified as protease and one missed cleavage was allowed. In database searches where acetylation of Lys residues was specified as additional variable modification, three missed cleavages were allowed to account for the loss of tryptic cleavage sites at acetylated Lys residues. Mass tolerances were set to 100 ppm for PMF searches and to 100 ppm (precursor ions) and 0.7 Da (fragment ions) for MS/MS ion searches. The minimal requirement for accepting a protein as identified was at least one peptide sequence match above identity threshold in addition to at least 20% sequence coverage in the PMF.

Where indicated, endoproteinase AspN was used as alternative protease for PTM mapping. For this purpose, excised gel spots were processed as above, but incubated with 0.8 ng AspN (sequencing grade, Roche, 11054589001) in 0.1% (*w*/*v*) octyl β-d-glucopyranoside (OGP), 5 mM Tris-HCl, pH 8.0 for 20 h at 37 °C. The amount of AspN was empirically adapted to minimize autolysis of the protease while still ensuring appropriate digestion of the substrate. In the corresponding database searches, AspN was specified as protease cleaving N-terminally of Asp and Glu residues, and up to three missed cleavage were allowed. To sequence large AspN-derived peptides (>3000 Da) by MALDI-MS, MS/MS experiments were performed from standard α-cyano-4-hydroxycinnamic acid (CHCA) dried droplet preparations using an UltrafleXtreme MALDI-TOF-TOF mass spectrometer (Bruker Daltonik). Peptide sequencing results were confirmed by electrospray LC-MS/MS using a Synapt G2-S quadrupole time-of-flight mass spectrometer equipped with ion mobility option (Waters Corporation, Milford, MA USA) as described previously [51].

### 2.10. Cryoimmuno Electron Microscopy

Immunogold labeling of cryosections was performed as described [52] on optic nerves of postnatal day 75. Antibody was specific for acetylated α-tubulin (1:1000, monoclonal mouse, Sigma, T 6793) and was detected by incubation with rabbit anti-mouse IgG secondary antiserum (1:200, Rockland Immunochemicals, Limerick, PA USA, #110-4102) which was visualized with protein A-gold (10 nm) (CMC, Utrecht, The Netherlands).

### 2.11. Estimation of Transfer Efficiency

Serial dilutions of myelin protein sample in sample buffer (150 mM Tris-HCl, pH 8.5, 2% (*w*/*v*) LDS, 10% (*v*/*v*) glycerol, 0.51 mM EDTA, 0.22 mM Serva Blue G250, 0.175 mM Phenol Red, 50 mM DTT) were prepared, incubated for 20 min at 40 °C and cleared from insoluble remains by centrifugation (1 min, 10,000× *g*, RT). Proteins were loaded to two precast NuPAGE 4–12% Bis-Tris Protein Gels (1 mm, 12 wells, Thermo Fischer Scientific). Protein load was 10 µg, 5 µg, 2.5 µg, 0.63 µg, 0.31 µg, 0.16 µg, 0.08 µg, 0.04 µg. 4 µL Dual Color Protein Standard III (Serva) and 4 µL SeeBlue-Plus-2 (Thermo Fischer Scientific) were used as molecular weight marker. Electrophoresis, staining with colloidal Coomassie and near-infrared imaging was performed as described above. For comparison to standard immunoblot transfer, the parallel gel was transferred to PDVF membrane using the same settings and procedures but for 60 min. Imaging, antibody incubation, and immuno-detection was performed as described above. Primary antibodies were used as follows: Acetyl Lysine Antibody (1:500, polyclonal rabbit, Immunechem, IPC0380) for 2 days and anti-MBP (1:1000, monoclonal mouse, BioLegend, San Diego, CA USA, 836501) over night at 4 °C. Secondary antibodies were used as follows: anti-rabbit IRDye 800 (1:5000, polyclonal goat, Biomol, 611-132-122), anti-mouse IRDye 680 (1:5000, polyclonal goat, Thermo Fisher Scientific, 982289).

## 3. Results

### 3.1. A Multi-Level Experimental Design for PTM Screening by 2D Gel Immunoblotting

**Figure 1:**
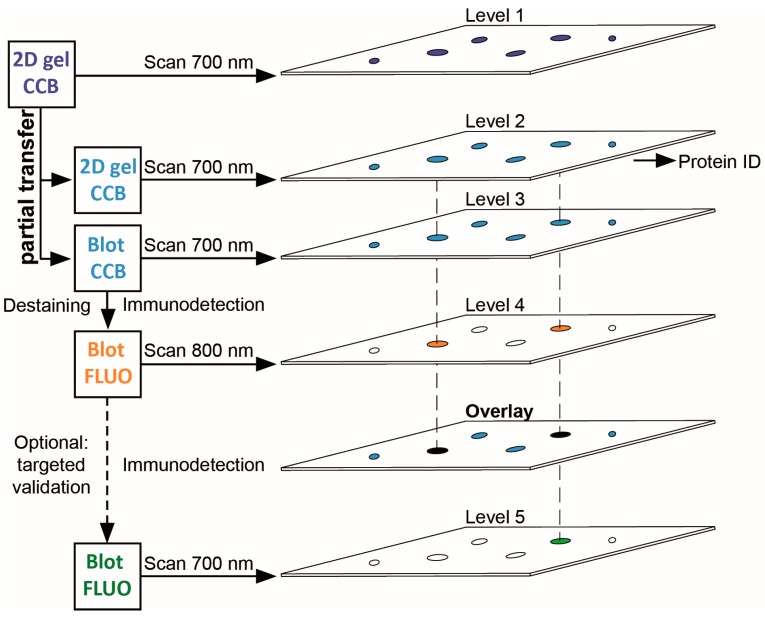
Workflow scheme for 2DE-based PTM screening. 2DE gels stained with colloidal Coomassie G-250 (CCB) are imaged using near-infrared fluorescence at 700 nm. Proteins are then partially transferred onto PVDF membranes. CCB-stained proteins remaining in the gel and CCB-stained proteins transferred to PVDF are visualized using the same imaging system. CCB stain is removed from the PVDF membrane and proteins with PTM are detected by PTM-specific antibodies using near-infrared fluorescence at 800 nm. Upon overlay in image analysis software, protein spots from the original gel corresponding to spots with PTM on the PVDF membrane can be subjected to protein identification and PTM site mapping. For immunoblot-validation of protein identification, antibodies specific for the respective protein can be used on the same PVDF membrane. Note that stripping of the PTM-specific antibody is typically not required when the 700 nm channel (formerly used for CCB detection) is employed.

In our workflow for PTM screening (Figure 1), proteins are separated by 2DE, stained with colloidal Coomassie, and the stained proteins are then partially transferred onto a PVDF membrane that can be subjected to immunodetection with PTM-specific antibodies. Importantly, the transfer of stained proteins allows for optical control of transfer efficiency and artifacts, and for reliable spot matching between gel and PVDF membrane. As a fraction of each protein is retained in the original gel, immunopositive proteins can be tracked back and mass spectrometrically identified from the same protein spot, thereby avoiding false assignment to abundant proteins migrating nearby. Subsequent validation of the protein identified can be readily included by its immunodetection in a second detection channel using the same PVDF membrane.

### 3.2. Immunodetection of Acetylated Myelin Proteins after Partial Transfer

**Figure 2:**
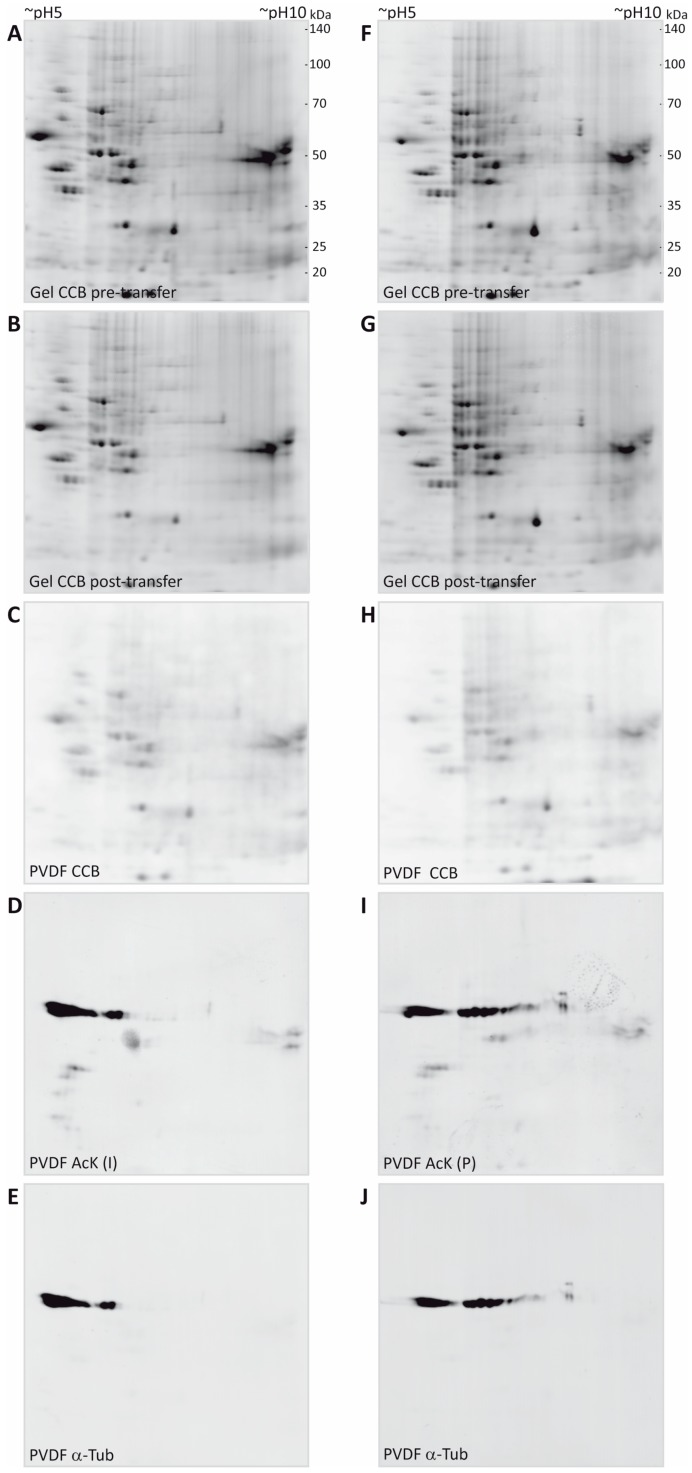
2DE separation and multi-level detection of myelin proteins. (**A**,**F**) Purified CNS myelin was separated using 2DE and stained with colloidal Coomassie. (**B**,**G**) Proteins remaining in the gel after partial transfer and re-stained with colloidal Coomassie. (**C**,**H**) Coomassie-stained proteins that were partially transferred onto PVDF membrane. (**D**,**I**) Acetylated proteins detected using two different antibodies specific for AcK (Immunechem, I; PTM biolabs, P). (**E**,**J**) Immunoblot-validation of spots constituted by α-tubulin.

To avoid loss of lysine acetylation, myelin was prepared in the presence of nicotinamide and trichostatin A for inhibition of protein deacetylases such as sirtuins and histone deacetylases, known to be contained in the myelin fraction. After separation by 2DE, proteins were denatured and precipitated within the gel by fixing in a solution containing ethanol and acetic acid. Importantly, the fixative did not contain any crosslinking reagents like formaldehyde or glutaraldehyde because they would interfere with protein transfer to the PVDF membrane and later protein identification by MS. Gels were stained according to a standard colloidal Coomassie protocol employing Coomassie Brilliant Blue G-250 (colloidal Coomassie Blue, CCB). The resulting background-free gels were imaged by near-infrared fluorescence (Figure 2A,F), leading to improved sensitivity, dynamic range, and signal-to-noise ratio in comparison to conventional densitometric detection (see Supplementary Figure S1 and [53,54]). Furthermore, this allowed us to use the same imaging system for capturing all images, including immunodetection, which facilitated spot matching by increasing the comparability within the experiment and by avoiding potential scaling differences.

Proteins from CCB-stained gels were blotted to PVDF membranes using wet transfer in a standard tank blot-system. Importantly, we optimized the duration of transfer in a way that all spots in the CCB-stained gels just become visible on the membranes and tried to retain the majority of the spot content in the original gel to facilitate subsequent identification by MS. By transferring stained spots, immediate visual control of transfer efficiency was possible. Overall, a transfer time of 13 min was found to be sufficient to partially transfer all spots to the PVDF membrane and no obvious loss of any protein spot was observed (Figure 2C,H). Due to a relatively high background level of CCB on the membrane and a somewhat more fuzzy spot appearance, the CCB signal on the PVDF membrane was used only for quality control of the transfer and spot matching, but not for quantification of transfer efficiency. Parallel experiments using serial dilutions of a myelin protein sample separated on 1D-gels using identical gel system and buffers, revealed a transfer efficiency of approximately 25%–50% for the 13 min partial transfer compared to a standard transfer of 60 min duration (Supplementary Figure S2).

CCB gel images and the CCB PVDF images were overlaid in the image analysis software Delta 2D by mapping protein spots. As frequently observed in electroblotting, transfer of proteins in the low molecular weight range appeared somewhat more efficient than that of proteins of higher molecular weight. As CCB stain was weak in the gels after transfer, gels were post-stained with CCB to facilitate later spot excision. Comparison of spot pattern and intensity in the gels before and after transfer confirmed that the optimized transfer conditions indeed retained most of the protein content in the gel (Figure 2B,G).

After imaging of the CCB-stained spots and the orientation marks in the 700 nm channel, the PVDF membranes were subjected to a destaining procedure to remove the CCB stain (but not the orientation marks), thereby avoiding potential interference of the CCB stain with immunodetection. The orientation marks were re-detected when the antibody signals were imaged in the 800 nm channel and allowed for exact manual overlay of scans, although only a few immunopositive spots were present. With the aim of showing the potential of our workflow even for quantitative comparison across separate 2D gel immunoblots, we used here the same myelin protein extract to compare two anti-acetyl-lysine (anti-AcK) antibodies from different suppliers (Immunechem, PTM Biolabs) in combination with secondary antibodies coupled to near-infrared fluorescent dyes. With the imaging system used in this study (Odyssey, Licor), secondary antibodies coupled to AlexaFluor680 (excitation 684 nm/peak emission 702 nm) or IRDye680 (ex: 680 nm/em: 692 nm) and AlexaFluor790 (ex: 783 nm/em: 803 nm or IRDye800CW (ex: 774 nm/em: 789 nm) can be used for multiplexing in the 700 nm channel and the 800 nm channel, respectively. We recommend to use the 800 nm channel for the first immunodetection (i.e., with the anti-AcK antibodies, Figure 2D,I), as residual signals from CCB may still be detectable in the 700 nm channel after the initial destain (not shown). After the second immunodetection for validation purposes, however, CCB staining is completely removed and the 700 nm channel is available for imaging of the second primary antibody. 

### 3.3. Identification of Potentially Acetylated Myelin Proteins

**Figure 3:**
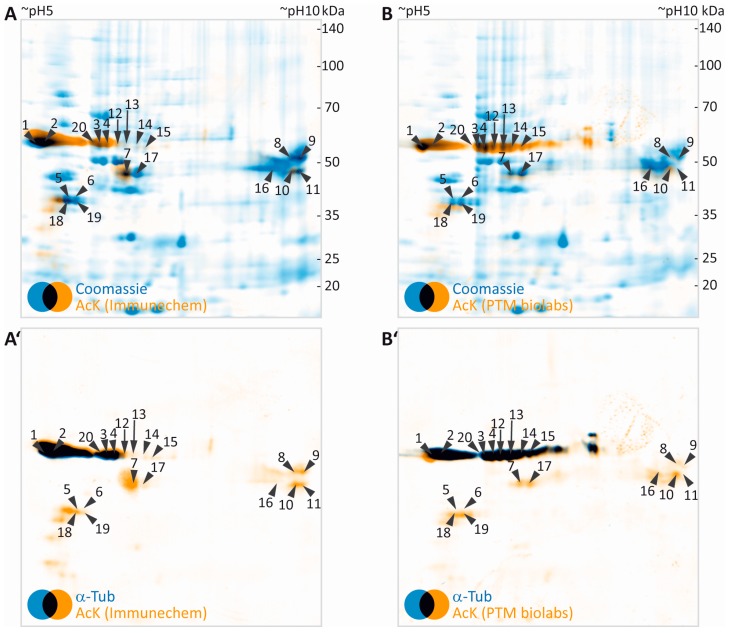
2DE-based screening for acetylated myelin proteins. (**A**,**B**) False-colored images of colloidal Coomassie-stained protein in gel (blue, images taken from Figure 2A,F) were superimposed to their corresponding images of AcK immunodetection (orange, images taken from Figure 2D,I). Spots with detectable AcK signal and visibility on colloidal Coomassie-stained gel were labelled with numbers and subjected to protein identification by MS (Table 1). (**A’**,**B’**) False- colored images of AcK immune detection (orange, images taken from Figure 2D,I) were overlaid to corresponding images of α-tubulin (blue, images taken from Figure 2E,J). Spot labels were transferred from panels A and B.

Both pan-AcK antibodies used in this study produced highly similar signal patterns, with a major AcK signal localized at an apparent molecular weight of ~50 kDa and an apparent isoelectric point of ~5–6 (Figure 2E,J). By using the orientation marks and the “implicit” warp function in the Delta2D software, AcK images were overlaid to CCB signal obtained from the initial CCB gel scan (Figure 3A,B). In total, 20 protein spots visible in CCB were immuno-positive for AcK signal (Figure 3A,B). The protein spots potentially containing acetylated proteins were excised and subjected to mass spectrometric protein identification. In the myelin protein fraction analyzed, the strongest AcK signal was obtained from spots containing α- and β-tubulin proteins (Table 1). A spot containing Septin 8 (SEPT8), a cytoskeletal myelin protein [55], was detected by the antibody used in Figure 3A (Immunechem), but was not clearly positive for AcK with the antibody used in Figure 3B (PTM Biolabs). The classical myelin protein 2′,3′-cyclic nucleotide phosphodiesterase CNP [56] was clearly identified in an AcK positive spot. Furthermore, ENPP6, an early oligodendrocyte protein [57] was identified in several spots. Some cytosolic proteins, GNB1, GNB2 and GLUL were identified in AcK positive protein spots. Furthermore, some potentially acetylated mitochondrial proteins were identified. This might account for co-purification of some mitochondria during myelin preparation [15]. For most of the eleven potentially acetylated proteins found in myelin, lysine acetylation was already described in other tissues (Table 1). 

Our workflow can be readily extended for validation of the identity of potentially acetylated proteins by using a second primary antibody specific for the respective protein. This is exemplified here for α-tubulin (Figure 3A’,B’). As indicated by black color derived from superimposing false-colored blue α-tubulin and orange AcK signals, the α-tubulin signal almost perfectly matched the major AcK signal obtained at 50 kDa/pH 5–6.

### 3.4. Tubulin Acetylation in Non-Compact Myelin

**Figure 4:**
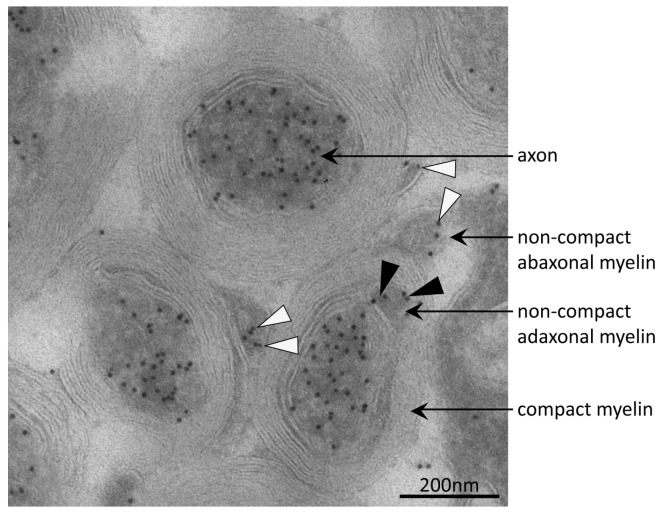
Immuno-electron micrograph detecting acetylated α-tubulin in axons and non-compact myelin. Acetylated microtubules were abundant in axons of mouse optic nerve as indicated by immuno-gold particles (black dots). In the adaxonal and abaxonal non-compact myelin compartment, acetylated microtubules were also present. Black and white arrow-heads point to corresponding immuno-gold particles in adaxonal and abaxonal non-compact myelin, respectively. On compact myelin, no immuno-gold was observed.

Acetylation at K^40^ of α-tubulin is known to mediate microtubule stabilization in the axons, the long projections of neuronal cells [67]. In order to rule out whether acetylated α-tubulin was detected in the myelin fraction only due to axonal contamination, we performed immuno-electron-microscopy. Optic nerve samples of young adult wild type mice were immuno-gold labeled for AcK^40^-α-tubulin (Figure 4). As expected, axons were highly decorated with immuno-gold, indicating a high percentage of acetylated microtubules in that cellular compartment. No immune-labeling was observed on compact myelin. Interestingly, the adaxonal and abaxonal non-compact myelin compartment containing cytosol with microtubules [68,69,70] were positive for AcK^40^-α-tubulin. 

### 3.5. Separation of Proteoforms: The Benefit of Including Internal Control Proteins

**Figure 5:**
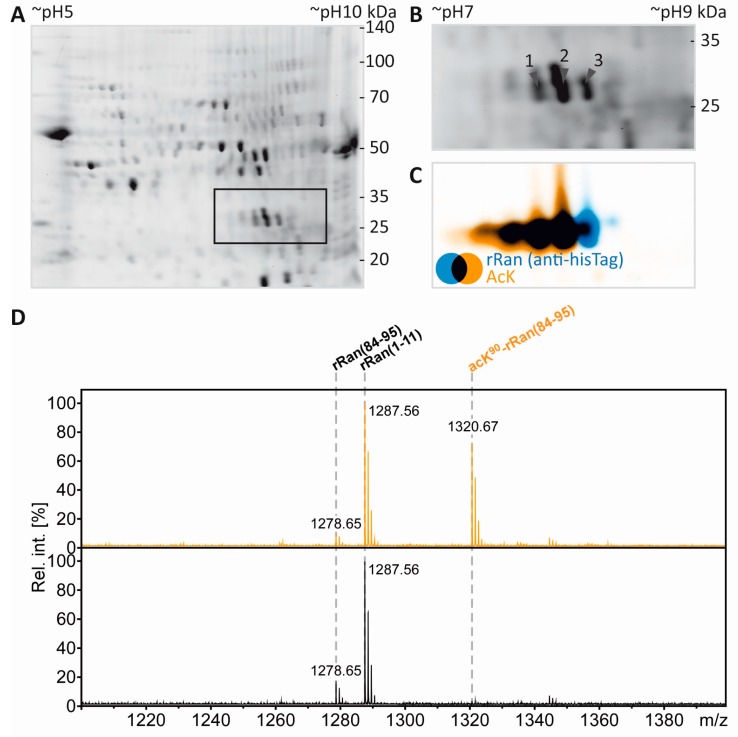

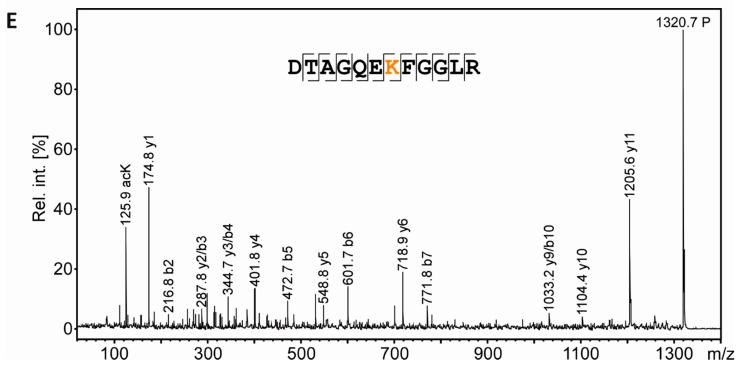
Separation and PTM mapping of rRAN proteoforms. **A**: Colloidal Coomassie-stained 2D-gel of myelin protein. Recombinant RAN (rRAN) specifically acetylated at K^90^ and non-acetylated rRAN spiked into the myelin sample immediately before IEF resulted in three additional protein spots. The box indicates the gel region shown in B. **B**: Gel region containing spots resulting from spiking of rRAN (labeled 1–3). **C**: Proteins were partially transferred onto PVDF-membrane and subjected to immunodetection using anti-AcK antibody (in orange) and anti-His-tag antibody (in blue), the latter detecting both, acetylated and non-acetylated, rRAN proteins. rRAN spots 1 and 2 show major acetylation as indicated by black color in overlay, whereas spot 3 contains rRAN, but only minor AcK signal. **D**: Zoom into the PMF spectra of rRAN peptides. The proteolytic peptide AcK^90^-rRAN(84–95) (*m/z* 1320.67) was prominent in spot 2 (upper panel), but virtually absent in spot 3 (lower panel). The corresponding non-acetylated peptide rRAN(84–95) (*m/z* 1278.65) was more abundant in spot 3 (lower panel) than in spot 2 (upper panel). The identity of all three peptides annotated was confirmed by mass spectrometric sequencing (only shown for AcK^90^-rRAN(84–95) in E. **E**: Sequencing of the proteolytic peptide AcK^90^-rRAN(84–95) by MS/MS. In the fragment ion mass spectrum, P denotes the precursor signal, and only b- and y-ions are labeled for the sake of clarity. On the basis of the conclusive N- and C-terminal ion series, acetylation was clearly assigned to K^90^. Mascot MS/MS ions score was 86 (identity threshold 31). Note that the signal at *m/z* 126 represents a signature immonium ion indicating the presence of AcK [71].

Acetylation of the free ε-amino group of lysine leads to the loss of a positive charge. In 2DE experiments this should be visible as a proteoform migrating at lower pH than the non-acetylated species. In the present experiments, tubulin as an established lysine-acetylated protein was expected to show this effect. However, the separation of the tubulin proteoforms was not sufficient, likely due to the high abundance and heterogeneity of tubulin. We therefore reasoned that our workflow would benefit from the inclusion of a recombinant protein and its lysine-acetylated counterpart as a kind of internal standard. Such a process control would provide a measure to ensure that the resolving power in the IEF dimension is sufficient for separation of lysine-acetylated from non-acetylated proteins and can also serve as positive control for mass spectrometric PTM mapping. To test the technical feasibility, 0.5 µg recombinant RAN (rRAN) and 0.5 µg rRAN with site-specific incorporation of *N*-(ε)-acetyl-lysine at K^90^ (AcK^90^-rRAN) was spiked into a myelin sample and subjected to our workflow. Three CCB-stained protein spots appeared in addition to the myelin spot pattern (Figure 5A,B), of which the two more acidic spots were found to contain a lysine-acetylated protein as revealed by incubation with pan-AcK antibody (Immunechem) after partial transfer (Figure 5C). All three spots were subsequently identified as rRAN using an anti-HisTag antibody as a second primary antibody for validation (Figure 5C). Acetylation of rRAN was verified and mapped to K^90^ after excision of the respective back-tracked spots from the original gel, in-gel digestion with endoproteinase AspN, and analysis by MS (Figure 5D,E). AspN was used as an alternative protease to be able to detect the same peptide with and without lysine acetylation, which would be impossible with trypsin not cleaving at acetylated lysine residues. In agreement with the immunoblot, the proteolytic peptide AcK^90^-rRAN(84–95) was prominent in the PMF spectrum obtained from spot 2 (Figure 5D) and its identity was unambiguously confirmed by mass spectrometric sequencing (Figure 5E). Accordingly, the corresponding peptide from non-acetylated rRAN was more abundant in spot 3, where the lysine-acetylated variant was virtually absent (Figure 5D). Although the distribution of the signature peptides between the spots indicates that the proteoforms are not fully separated, we consider the resolving power of our method as sufficient for studying lysine acetylation and most likely also other charge-modifying PTMs, such as phosphorylation of Ser/Thr/Tyr and citrullination of Arg.

### 3.6. Mass Spectrometric Validation of AcK^40^ in α-Tubulin

**Figure 6:**
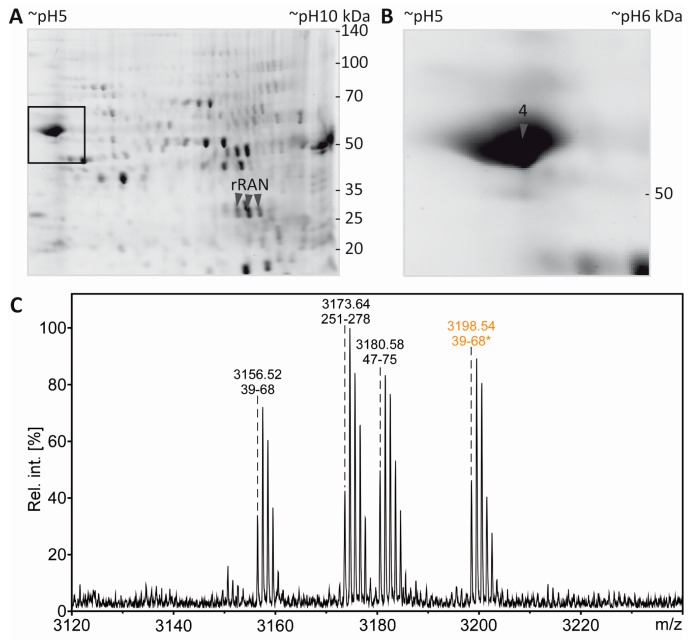

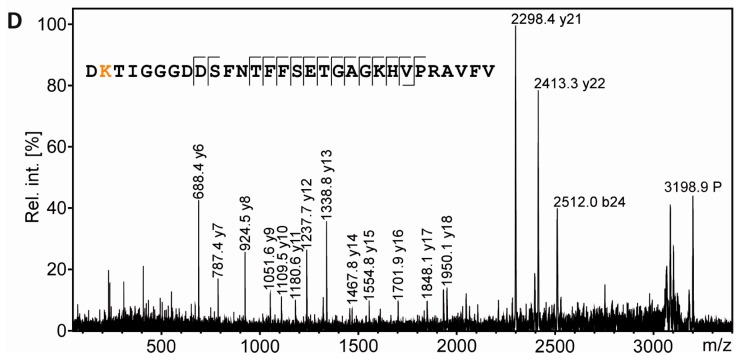
PTM mapping of α-tubulin. **A**: Colloidal Cooomassie-stained 2D-gel of myelin protein with spiked rRAN (same gel as in Figure 5). The box indicates the gel region shown in B. **B**: Spot 4 was excised for in-gel digestion using AspN. **C**: PMF spectrum of α-tubulin peptides. The zoom into the PMF spectrum shows prominent signals for the proteolytic peptide α-tubulin(39–68) without AcK (*m/z* 3156.52) and α-tubulin(39–68) with AcK (*m/z* 3198.54, marked with an asterisk and depicted in orange), along with signals for other AspN-derived α-tubulin peptides (amino acid sequences 251–278 and 47–75). **D**: Sequencing of the proteolytic peptide containing AcK^40^-α-tubulin(39–68) by MS/MS. In the fragment ion mass spectrum, P denotes the precursor signal, and only b- and y-ions are labeled for the sake of clarity. Mascot MS/MS ions score was 108 (identity threshold 33). Fragment ions were compatible with acetylation at K^40^, which was verified further by electrospray LC-MS/MS (Supplementary Figure S3).

From the same gel as shown in Figure 5, a spot was excised from the region known to be highly immunopositive for AcK and to contain α-tubulin (Figure 6A,B; label 4) according to the experiments described above. Like the spots containing the rRAN control proteins, the sample was processed by in gel digestion using AspN as alternative endoproteinase. In the PMF spectrum obtained from spot 4, signals were detected which corresponded to the AspN-derived peptide α-tubulin(39–68) in its unmodified and in its acetylated form, respectively (Figure 6C). Of note, this annotation was only possible when three missed cleavage sites were allowed, probably reflecting a somewhat inferior performance of AspN in comparison to trypsin. Sequencing of AcK^40^-α-tubulin(39–68) by MALDI-TOF-MS/MS in principle supported K^40^ as the acetylation site (Figure 6D), although the lack of an N-terminal fragment ion series prevented an unambiguous assignment. We thus applied electrospray LC-MS/MS as a complementary technique, because large peptides typically fragment more efficiently with this method due to multiple charging. Thereby, conclusive N- and C-terminal fragment ion series were obtained, confirming the identity of AcK^40^-α-tubulin(39–68) and two other AcK^40^-containing α-tubulin peptides (Supplementary Figure S3).

## 4. Discussion

We describe a 2DE-based low-tech method for PTM screening that is easily adaptable to any kind of antibody-detectable protein modification. The entire workflow is fast and easily accessible: within the timeframe of three to four working days a complete screening experiment can be performed in nearly any biochemical laboratory. Protein staining by CCB is cheap compared to other commercially available fluorescent stains like SYPRO ruby, Krypton or Flamingo, but provides similar quality when imaged with a near-infrared fluorescence detection system [53,54].

Classically, staining of protein spots on PVDF membranes after transfer is used to visualize protein spot patterns and several staining protocols are available for that purpose. Options for post-transfer protein staining include the low-sensitivity stains Ponceau S and Coomassie, as well as Fast-Green FCF and Direct Blue 71 that are described to be more sensitive [54,72,73]. Following the method of Welinder and Ekblad [74], we have tried the application of Coomassie for staining of PVDF membranes after immunodetection, which comes with the advantage that potential interference of the dye with antibody binding or signal detection is excluded. However, even after testing several adaptations, the sensitivity of this method was not sufficient for reliable spot matching in our hands (data not shown). As a straightforward workaround for visualizing the spot pattern on PVDF membranes, we describe here the transfer of proteins from CCB-stained gels, allowing full visual control of transfer efficiency and artifacts. Accordingly, all imaging steps during the experiment can be performed with the same imaging system, providing maximal step-to-step comparability. Some precautions are required for transfer of gels after staining. The fixative must not contain any cross-linking substances, e.g., formaldehyde or glutaraldehyde. The fixation principle used in this study is by in-gel protein denaturation/precipitation, similar to the procedure recommended for the fixation of IEF gels prior to transfer to the second dimension SDS-PAGE (see for example the Novex IEF gel manual, Thermo Fisher Scientific). For electroblotting to PVDF membranes, gels need to be equilibrated in SDS- or LDS-containing buffer to mobilize proteins, similarly as described for transfer to nitrocellulose membranes [75]. 

We present our technique as a comparative approach. Usage of image analysis software like Delta2D (Decodon) enables warping of parallel gels. The option of matching across separate 2D-gels makes our workflow attractive for quantitative comparison of PTMs in many experimental settings, e.g., mutant vs. wildtype or treatment vs. control. Since transfer efficiency critically affects the reliability of comparisons and is influenced itself by choice of equipment and buffers, we suggest to perform comparative approaches as parallel experiments only. Here, we have exemplified this potential by comparing two different antibodies for the detection of lysine-acetylated proteins. In myelin, we identified eleven potentially acetylated proteins represented in 20 protein spots. For most of them, acetylation was described previously in other tissues (Table 1). Compared to cellular subfractions like mitochondria [43,59], a relatively low number of proteins within the myelin fraction were found to be acetylated. Among them were a number of proteins with described mitochondrial localization that might originate from mitochondrial contamination during myelin preparation [15]. Some myelin proteins that were described as immunopositive for acetyl-lysine antibody, e.g., MBP or MOG [15,43] were not identified in the present screen, which is probably due to poor representation of these very basic or hydrophobic proteins in 2DE [15]. 

At least three proteins identified in AcK-immunopositive spots belong to the cytoskeleton: the cytoskeletal protein SEPT8 [55], the microtubule-associated CNP [76] and the microtubule protein α-tubulin. Acetylation stabilizes microtubules [67]. In CNS, acetylated microtubules are mainly located in axons [77] in which they facilitate the long term maintenance of neuronal integrity [78]. For the myelin compartment, tubulin acetylation was not described before. The theoretical possibility that acetylated α-tubulin in the myelin fraction is only due to axonal contamination is ruled out by our observation of acetylated microtubules within the non-compact myelin compartment by immuno-electron microscopy. This finding implicates, that stable oligodendroglial microtubules are present within the cytosolic channels through myelin connecting the oligodendrocyte soma with the inner tongue [4]. After complete myelination, myelin is highly stable [79,80], but a permanent turnover of myelin membranes is required for myelin maintenance [81]. Furthermore, metabolic supply to the axons has recently emerged as a key feature of oligodendroglial function [82,83,84,85]. These functions of myelin beyond passive electrical isolation probably involve transport processes through the myelinic channels, most likely along microtubules. Disturbances in oligodendroglial microtubule dynamics cause demyelination and axonal degeneration [86]. Thus, we hypothesize, that regulation of cytoskeleton dynamics in myelin by acetylation/deacetylation might serve adaptations essential for axonal integrity.

The most abundant acetyl-lysine signal was mapped to α-tubulin. Acetylation of α-tubulin at K^40^ was described first by epitope mapping [87]. Although known since decades, most MS-based acetylome studies lack identification of α-tubulin AcK^40^ [33,88]. This is most probably due to usage of trypsin as single protease and inhibition of tryptic cleavage at acetylated lysines [89]. Digestion of mouse α-tubulin at AcK^40^ with trypsin results in the missed cleavage at that position and thus in an unusually large tryptic peptide (6316 Da), that is not readily accessible by standard, collision-induced dissociation-based mass spectrometric sequencing. In studies using combinations of proteases, AcK^40^ containing peptides were identified [34,90]. However, assignment of AcK^40^ is often compromised by low identification and PTM scoring or by consideration of cleavage at AcK^40^ even when unlikely, and should therefore be viewed with some caution. This motivated us to test an alternative protease, and we established the use of AspN for comparative PTM mapping of rRAN and AcK^90^-rRAN, recombinant proteins that we have spiked into myelin samples as internal controls for proteoform separation. Finally, the same strategy was successful for specific identification of AcK^40^ in the proteoform co-detected by Pan-AcK and α-tubulin antibodies.

One potential limitation of our approach is the fact that immunodetection is often still more sensitive than the mass spectrometric analysis, at least when 2DE is directly interfaced to conventional MALDI-TOF mass spectrometry without prior separation at the peptide level. For example, almost all spots immunopositive for α-tubulin were mass spectrometrically identified as α-tubulin, but in three spots ENPP6 was identified instead (Table 1). Thus, the AcK signal in those spots might also be derived from α-tubulin and not from the abundant, co-migrating protein. This underscores the need for validation and mapping of the site of modification by alternative methods, including antibody-based enrichment of modified peptides and more sophisticated MS approaches. 

By using antibodies directed to PTMs other than acetylated lysine, the method described here can be easily adapted to any PTM for which specific antibodies are available (e.g., nitrosylation, citrullination, and malonylation). Furthermore, we see the possibility to use our 2DE partial immunoblotting method for immunoproteomics. In serological proteome analysis (SERPA), a major problem is alignment of 2D protein spots with the antigen pattern immunopositive after probing the membrane with patient serum [46]. Identification of bacterial antigens or autoantigens was often complicated due to different complexity levels in protein and immunopositive pattern or high antigenicity despite low protein abundance, interfering with post-experimental alignment [91,92,93]. In order to circumvent this, other attempts include the application of internal markers [94], additional staining steps [91], and alternative usage of protein arrays [95,96] or Luminex assays [97] based on recombinant proteins. 2DE partial immunoblotting as described here could complement screening approaches for linear protein antigens without bias to known antigens. 

## 5. Conclusions

We provide a method to identify post-translationally modified proteins that does not require advanced sample preparation and mass spectrometric approaches. Indeed, the combination of 2DE, colloidal Coomassie staining, partial immunoblotting, signal detection in multiple channels and conventional mass spectrometry allows the straightforward screening of PTMs in a sample of interest. Considering that the deacetylase SIRT2 is very abundant in myelin [15], we hypothesize that acetylation/deacetylation may be important for regulating the activity of myelin proteins. Indeed, we found multiple potentially acetylated myelin proteins, including the cytoskeletal protein SEPT8 [55] and the cytoskeleton-associated CNP [76]. Yet, the most abundant acetylated protein in myelin is tubulin. Importantly, the stability of microtubules (i.e., tubulin polymers) is controlled by acetylation [67]. Together, the myelin acetylome appears to be mainly constituted by cytoskeletal and cytoskeleton-associated proteins. We believe that our method holds great promise beyond identifying lysine-acetylated proteins or other PTMs for which specific antibodies are available. For example, adaptations of our workflow may well enable the identification of yet unresolved auto-immune antigens or host-pathogen interactions. 

## Figures and Tables

**Table 1:** Identification of lysine-acetylated myelin proteins.

Gene Name	UniProtKB Accession	Protein Name	MW (Da)	CpI	Spot	PMF Score Gel A/Gel B	PMF Coverage [%] Gel A/Gel B	No. of Peptides Sequenced Gel A/Gel B	Cumulative MS/MS Score Gel A/Gel B	References for Previous Identification of Lysine Acetylation
**Atp5b**	P56480	ATP synthase subunit beta, mitochondrial	56,265	5.1	**1**	212/224	66/58	3/4	227/231	[33,34,43,58,59,60,61,62]
**Cnp**	P16330	2′,3′-cyclic-nucleotide 3′-phosphodiesterase	47,493	9.1	**8**	72/61	37/26	2/-	94/-	[34,58,59,60,63]
					**9**	218/147	55/39	3/2	262/106	
					**16**	246/147	59/46	4/2	353/107	
**Enpp6**	Q8BGN3	Ectonucleotide pyrophosphatase/phosphodiesterase family member 6	50,928	6.9	**13**	-/67	-/29	-/1	-/29	[34,63]
					**14**	72/50	21/15	2/1	58/30	
					**15**	102/-	32/-	3/1	77/30	
**Glul**	P15105	Glutamine synthetase	42,834	6.6	**7**	127/143	28/32	2/4	61/183	[34,43,59,60,63,64,65]
					**17**	163/197	50/52	4/4	281/236	
**Gnb1**	P62874	Guanine nucleotide-binding protein G(I)/G(S)/G(T) subunit beta-1	38,151	5.6	**5**	73/98	27/34	-/2	-/43	[34]
					**6**	90/130	27/41	-/3	-/102	
**Gnb2**	P62880	Guanine nucleotide-binding protein G(I)/G(S)/G(T) subunit beta-2	38,048	5.6	**18**	132/92	48/32	2/1	67/29	-
					**19**	102/167	44/55	3/3	130/122	
**Got2**	P05202	Aspartate aminotransferase, mitochondrial	47,780	9.1	**10**	93/105	34/35	-/1	-/33	[33,34,43,58,59,60,61,62,65]
					**11**	107/-	39/-	3/-	50/-	
**Sept8**	Q8CHH9	Septin-8	50,123	5.7	**20**	96/98	33/33	1/-	28/-	[34,65]
**Tuba1a**	P69369	Tubulin alpha-1A chain	50,788	4.9	**1**	132/83	51/36	4/4	73/130	[33,58,66]
					**3**	70/90	24/33	1/1	42/41	
					**20**	61/60	21/20	1/2	42/60	
**Tuba1c**	P68373	Tubulin alpha-1C chain	50,562	5.0	**4**	76/76	33/33	1/1	36/36	[33,58,59,66]
**Tubb4a**	Q9D6F9	Tubulin beta-4A chain	50,010	4.8	**1**	105/74	43/26	2/-	42/-	[33,58,59,66]
					**4**	88/147	26/50	-/1	-/32	
					**12**	-/141	-/40	-/1	-/27	
					**13**	-/147	-/46	-/1	-/28	

## Supplementary Materials

The following are available online at www.mdpi.com/2227-7382/5/1/3/s1. Figure S1: Detection sensitivity of different scanning methods. Figure S2: Estimation of transfer efficiency. Figure S3: Confirmation of K^40^ as acetylation site in α-tubulin by LC-MS/MS.

## Abbreviations

The following abbreviations are used in this manuscript

PTM	post-translational modification
2D	two-dimensional
PVDF	Polyvinylidene difluoride
SERPA	serological proteome analysis
CNS	central nervous system
MS	mass spectrometry
TBS	Tris buffered saline
PBS	Phosphate buffered saline
IEF	isoelectric focusing
CCB	colloidal Coomassie
AcK	acetylated lysine
SDS	Sodium dodecyl sulfate
LDS	Lithium dodecyl sulfate
2DE	2D gel electrophoresis
SDS-PAGE	sodium dodecyl sulfate polyacrylamide gel electrophoresis
