# Supplementary material for: Partial Immunoblotting of 2D-Gels: A Novel Method to Identify Post-Translationally Modified Proteins Exemplified for the Myelin Acetylome
Source: Proteomes. 2017 Jan 12;5(1):3. doi: 10.3390/proteomes5010003 (PMC5372224; doi:10.3390/proteomes5010003)
Supplement: Supplementary file 1 [file proteomes-05-00003-s001.pdf]

Supplementary Materials:

Partial Immunoblotting of 2D-Gels: A Novel Method to Identify Post-Translationally Modified Proteins Exemplified for the Myelin Acetylome

Kathrin Kusch, Marina Uecker, Thomas Liepold, Wiebke Möbius, Christian Hoffmann, Heinz Neumann, Hauke B. Werner and Olaf Jahn

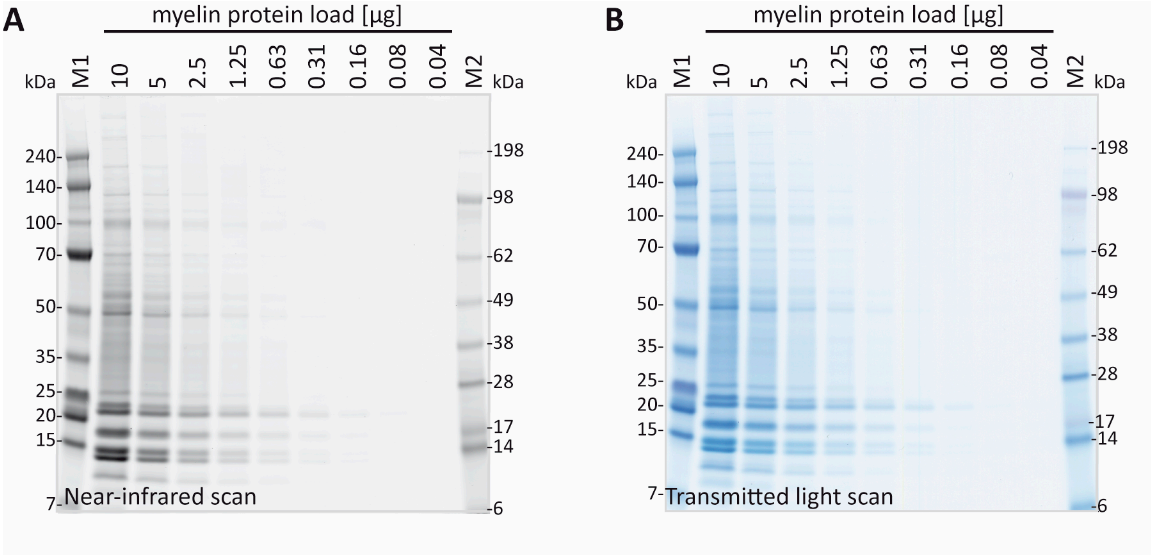

Figure S1. Detection sensitivity of different scanning methods. Serial dilutions of a myelin sample were separated in a 1D gel and stained with colloidal Coomassie (CCB). The gel was imaged by near-infrared detection (**A**) and by visible transmitted light scanning (**B**). Near-infrared detection was found to have a higher dynamic range and an approximately two-fold higher sensitivity.

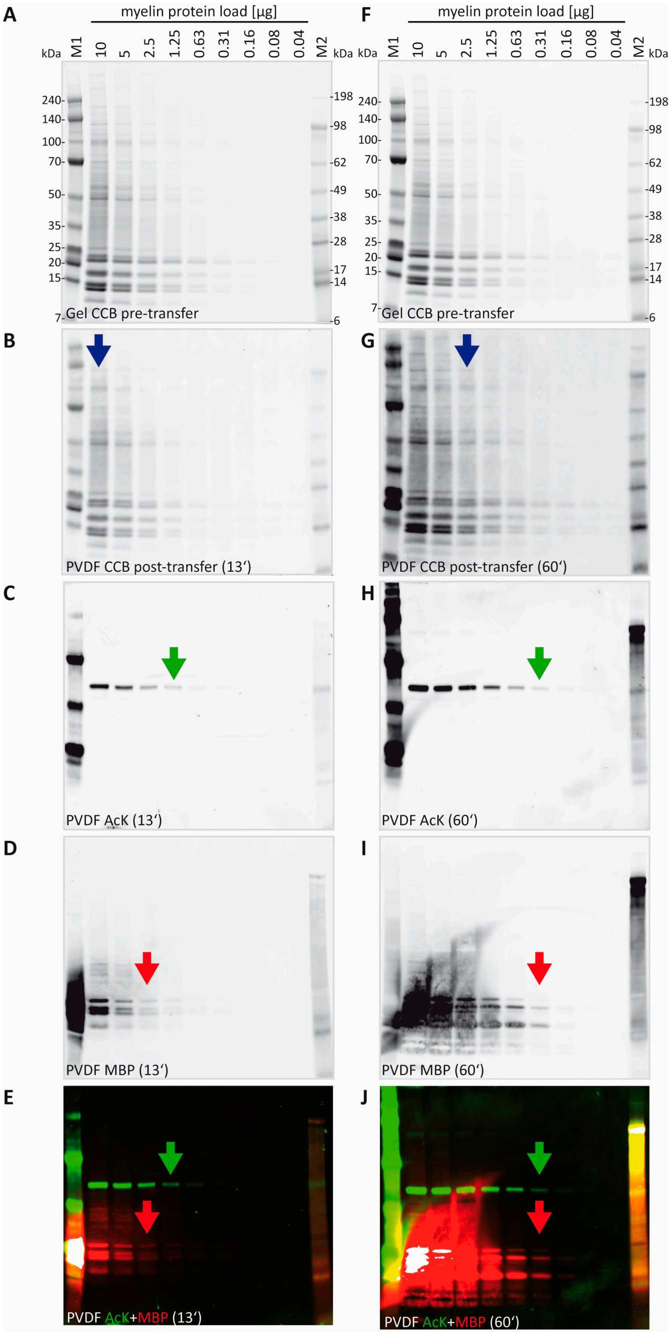

Figure S2. Estimation of transfer efficiency. (**A**,**F**) Serial dilutions of a myelin sample were separated in two parallel 1D gels and stained with colloidal Coomassie (CCB).(**B**,**G**) PVDF membrane with CCB stained proteins after partial transfer for 13 min **(B)** or standard transfer for 60 min **(G)**. Lanes indicated by blue arrows (10 µg protein in (**B**) and 2.5 µg protein in (**G**)) appeared similar in CCB staining intensity. (**C**,**H**) Acetylated proteins detected using an antibody specific for AcK (Immunechem). Lanes indicated by green arrows appeared similar in AcK immunofluorescence intensity (1.25 µg in (**C**) and 0.31 µg in (**H**)). Note that image acquisition and processing parameters were chosen for optimal display of the most prominent tubulin band, leaving other less intense signals (e.g. for acetylated MBP) undetected. (**D**,**I**) Myelin basic protein (MBP) detected using an antibody specific for MBP (BioLegend). Lanes indicated by red arrows appeared similar in MBP immunofluorescence intensity (2.5 µg in **D** and 0.31 µg in **I**). **(E**,**J)** False-colored overlay images of AcK (false colored in green) and MBP (false colored in red) immunodetections as shown separately in (**C**,**D**) or (**H**,**I**)**,** respectively. White color indicates signal saturation in the respective lane. Protein standard used were M1: Dual color Protein Standard III (Serva) and M2: SeeBlue-Plus-2 (Thermo Fischer Scientific, Waltham, MA USA).

A

No.	Peptide	Sequence	[M+H]+ obs.	[M+H]+ calc.	ΔM (ppm)	Score	b-y	RMS (ppm)
1	39-68	DKTIGGGDDSFNTFFSETGAGKHVPRAVFV	3198.5425	3198.5490	-2,04	6.50	17	6.97
2	39-45	DKTIGGG	689.3450	689.3465	-2.16	6.12	8	10.30
3	33-45	DGQmPSDKTIGGG	1320.5703	1320.5736	-2.56	6.03	4	8.49

B

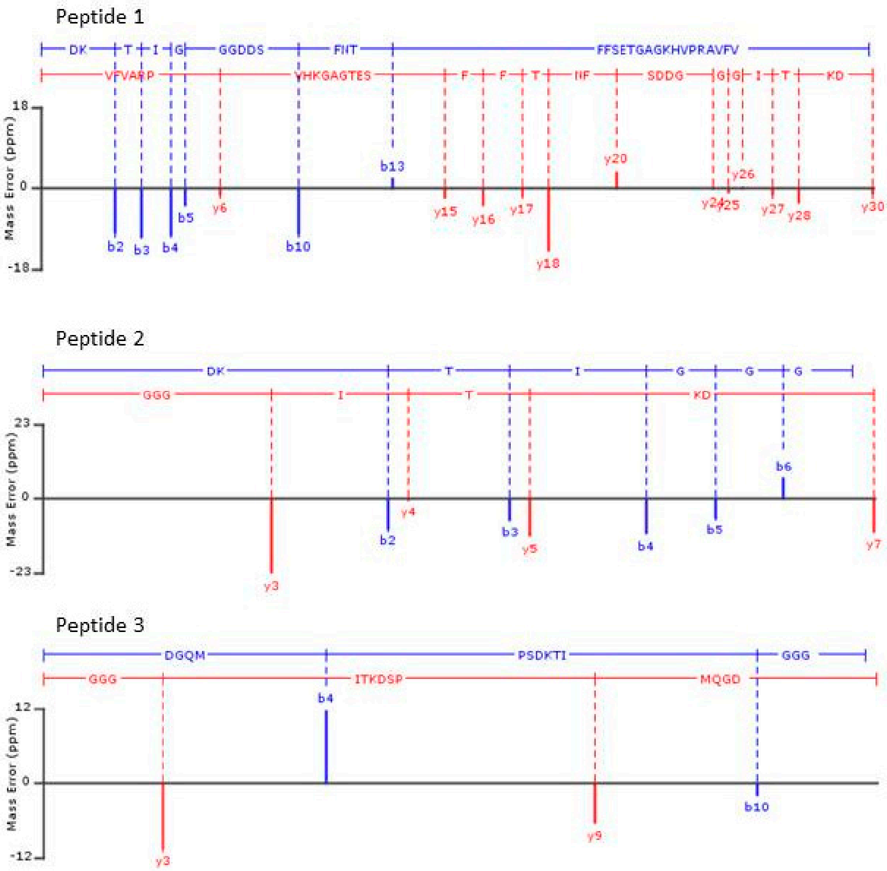

Figure S3. Confirmation of Lys-40 as acetylation site in α-tubulin by LC-MS/MS. (**A**) Table with identification details for the detected AcK[40]-containing α-tubulin peptides. Columns show from left to right: number of peptide, numbering of amino acids according to the sequence of α-tubulin (UniProtKB accession P68369); peptide sequence (m, oxidized Met); observed and calculated mass of the singly protonated peptide; peptide mass deviation in ppm; PLGS score; number of b–y fragment ions; root mean square (RMS) fragment mass deviation in ppm. (**B**) Fragment ion mass spectra reconstructions on the basis of ion mobility-powered data-independent acquisition data (HDMS^{E}). Only b- and y-ions are labeled for the sake of clarity, and fragment ion mass deviation is shown. For peptide 1 also sequenced by MALDI-MS, acetylation was clearly assigned to K40 on the basis of the conclusive N-terminal ion series down to b2.
